# Beta-Myrcene as a Sedative–Hypnotic Component from Lavender Essential Oil in DL-4-Chlorophenylalanine-Induced-Insomnia Mice

**DOI:** 10.3390/ph17091161

**Published:** 2024-09-01

**Authors:** Luge Chen, Yingwei Liu, Dawei Xu, Na Zhang, Yong Chen, Jin Yang, Lijuan Sun

**Affiliations:** 1 National & Local Joint Engineering Research Center of High-Throughput Drug Screening Technology, Hubei University, Wuhan 430062, China; clg990522@163.com (L.C.); lyw15972985208@126.com (Y.L.); xudawei1372023@163.com (D.X.); zhna18@163.com (N.Z.); cy101610@hubu.edu.cn (Y.C.); 2 School of Traditional Chinese Medicine, Hubei University for Nationalities, Enshi 445000, China

**Keywords:** lavender essential oil, beta-myrcene, insomnia, serotonergic synaptic pathway

## Abstract

With the increasing prevalence of insomnia-related diseases, the effective treatment of insomnia has become an important health research topic. Lavender (*Lavandula angustifolia* Mill.) essential oil (LEO) is a commonly used medicine for the treatment of insomnia and neurological disorders. However, neither the active components nor its sedative–hypnotic mechanism have been fully discovered. This study aimed to screen the main active terpenes and discover the possible mechanism of LEO through network pharmacology in the treatment of insomnia-related diseases, as well as to verify our hypothesis in insomnia mice. The results showed that, in LEO’s 15 potential active ingredients, beta-myrcene had strong sedative–hypnotic effects through the serotonergic synaptic pathway according to the network pharmacological prediction. Further, PCPA(DL-4-chlorophenylalanine)-induced insomnia mice were treated with beta-myrcene for one day or seven days. The quiet state of insomnia mice was increased effectively, and the hypnotic effect was enhanced by anaobarbital sodium by prolonging sleep duration, decreasing sleep latency, and increasing the rate of falling asleep. Beta-myrcene reduced the damage to hypothalamic neuron cells induced by PCPA and increased neurotransmitter levels of GABA, 5-HT, and Glu in the serum and hypothalamus of insomnia mice. Meanwhile, beta-myrcene exerted an improvement in insomnia by upregulating relevant genes and protein expression in the serotonergic synaptic pathway. These results support the merit of the sedative–hypnotic activity of LEO. Beta-myrcene, a terpene in LEO, may be the main source of its sedative–hypnotic properties. It may serve as a good potential compound in future clinical studies on coping with insomnia.

## 1. Introduction

Insomnia is a sleep circumstance marked by recurring and ongoing challenges with falling and staying asleep, leading to inadequate sleep quality. Chronic insomnia may result in emotional issues like depression and anxiety, weaken the immune system, and increase the probability of developing hypertension, coronary heart disease, and depression, thus having a significant impact on overall health [1,2]. Insomnia is increasingly common in today’s society, driven by the escalating pressures of daily life and academic expectations. Common medications for insomnia are benzodiazepines, non-benzodiazepines, sedative antidepressants, and others [3]. Their continuous consumption may result in negative effects, including dependence, tolerance, withdrawal symptoms, and rebound effects. Researchers have long sought to find safer and reduced-side-effect compounds in natural plants. A growing quantity of scholars are interested in researching new and secure supplemental or alternative medicines for treating insomnia.

Lavender (*Lavandula angustifolia* Mill.) is a natural sterile hybrid and has been used as a significant Chinese herbal medicine (CHM) in many regions since ancient times [4,5]. It has been shown that a combination of essential oils containing LEO can alleviate PCPA-induced insomnia, reduce spontaneous activity, and increase 5-HT and GABA levels in the brain in mice [6]. LEO also can synergize with sodium pentobarbital to enhance sleep rates and reduce anxiety as well as sleep disturbances induced by caffeine injection in mice [7]. The main components of LEO are mainly terpenes and sesquiterpenes [8]. Beta-myrcene is one of the main components of *Myrtus Communis* essential oil (Morocco), which has antibacterial activity in vivo and in vitro [9]. In the study of ancient bayberry’s resistance to stress, beta-myrcene is the main metabolite related to its resistance [10]. But there is no research on the effects of beta-myrcene on sleep. It was found that 3-carene can improve the duration of sleep in mice and decrease sleep latency [11]. Subsequent molecular analysis revealed that 3-carene acted on GABAA-BZD receptors to boost GABAergic synaptic responses by extending the decay time constant of the spontaneous inhibition of postsynaptic currents (sIPSCs) in a dose-dependent manner. Moreover, rats’ REMS can be markedly increased by *α*-pinene, and it can also modulate their circadian rhythm following inhalation [12]. Additionally, oral (-)-*α-*pinene treatment dramatically expanded the duration of NREMS sleep-phase syndrome and diminished sleep latency, while not affecting REMS duration [13]. Furthermore, (-)-*α*-pinene had a regulatory function through direct binding to GABAA-BZD receptors, which boosted the receptor-mediated GABAergic synaptic response and performed a role in inducing sleep. In a pentobarbital-induced sleep experiment, it was reported that β-caryophyllene reduced the time required for mice to fall asleep, thereby prolonging their sleep duration [14].

Currently, there are no studies reported about the individual terpenes of LEO and their effects on alleviating insomnia. This paper focused on examining the anti-insomnia activity of the active ingredients of LEO first, specifically the main terpenes and sesquiterpenes. The anti-insomnia ingredients and signaling pathways were screened by network pharmacology. Furthermore, since PCPA-treated ICR mice serve as an insomnia model [15], we chose this model to further conduct a relevant investigation in vivo.

## 2. Results

### 2.1. Selection of Targets and Network Analysis

**Figure 1:**
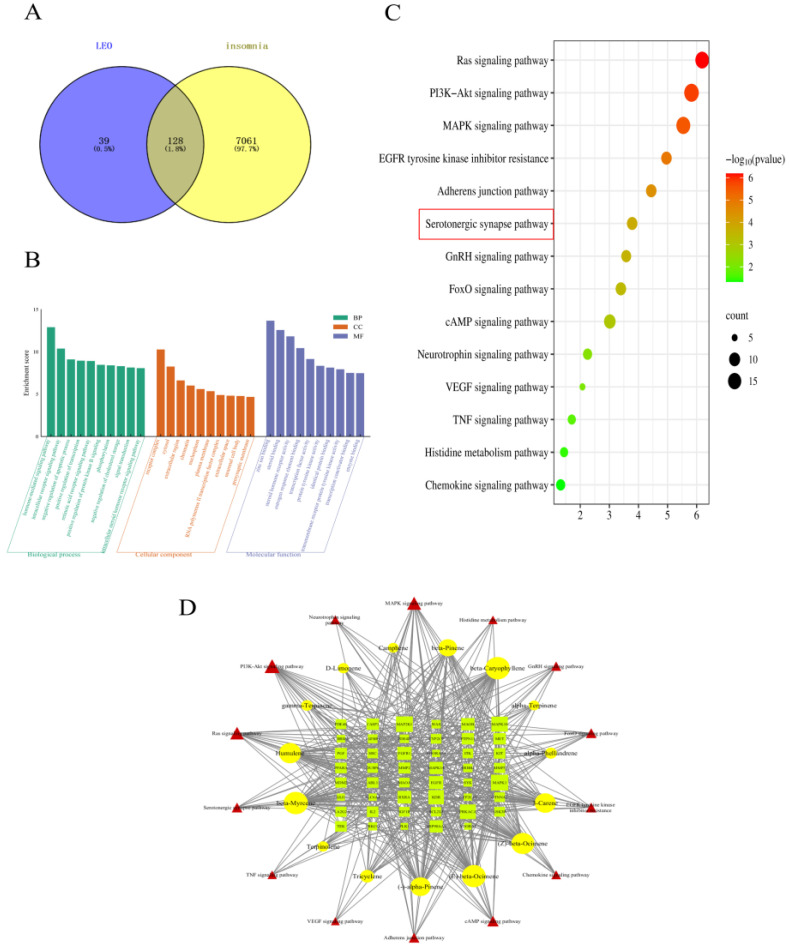
LEO “active constituents-sleep” target-related network figures. (**A**) Crossed gene target graph. (**B**) Gene Ontology enrichment analysis. (**C**) KEGG pathway enrichment analysis. (**D**) ”Active constituents-Potential targets-Pathways” network of LEO.

A total of 167 LEO active gene targets in 15 terpenes were obtained from the Swiss Target Prediction database combined with the PharmMapper database. In addition, a total of 7189 disease-related targets were obtained from the GeneCards database, OMIM database, and TTD database search. These corresponding targets obtained from the screening of active components of LEO were imported into Venny2.1.0 with the relevant targets of sleep disorders, and 128 intersecting targets of the possible effects of LEO to improve sleep were obtained, and the results are shown in Figure 1A. These shared targets represent 76.65% of the active ingredient target library of LEO, indicating that the 15 active ingredients of LEO have the potential to improve insomnia.

### 2.2. GO and KEGG Enrichment Analyses

The 128 targets shared by LEO active ingredients and sleep regulation were imported into the DAVID6.8 online platform for GO function and KEGG pathway enrichment analysis. The results showed that a total of 261 BP, 41 CC, and 97 MF were obtained by GO functional analysis enrichment at *p* < 0.05. Based on the negative logarithm of the *p*-value enrichment fraction, the above three types of data were ranked in descending order, and the top 10 in each group were selected to draw a bar chart on the microbiology letter platform (Figure 1B). A total of 103 pathways of action were obtained from the results of KEGG pathway analysis, and the pathways related to the regulation of the nervous system were screened according to the literature, arranged in descending order based on the enrichment scores, and, subsequently, enrichment bubble diagrams were plotted (Figure 1C). The results showed that LEO mainly acted on cellular components such as the cytoplasm, nucleus, and cell membrane, thus affecting gene expression regulation, cell proliferation, apoptosis, and a variety of molecular functions, including proteins, enzymes, ATP, and DNA binding, as well as potentially exerting the therapeutic effects of LEO on insomnia through the conduction of the serotonergic synapse signaling pathway and cAMP signaling pathway, among others.

### 2.3. Screening the Results of the Construction of the “Active Ingredients-Potential Targets-Pathways” Network for LEO

A total of 14 signaling pathways with relevance to the two sleep disorders and the targets associated with these pathways were combined in the KEGG enrichment results. The network visualization was constructed with the selected 15 LEO active ingredients imported into Cytoscape 3.7.2 software with 76 nodes and 497 edges (Figure 1D). Through the analysis of the NetworkAnalyzer tool in Cytoscape 3.7.2, beta-myrcene and beta-caryophyllene are the most prominent active ingredients in regulating sleep among the LEO constituents. Serotonergic synapses signaling pathway and cAMP signaling pathway may be the main pathways involved in the regulation of sleep disorders.

Based on the above network pharmacological results, beta-myrcene is the main component, with its content of LEO being 5.641%, which is higher than beta-caryophyllene [16]. Hence, beta-myrcene was selected as the main active ingredient, and the serotonergic synaptic pathway was selected as the main research pathway according to KEGG results, and important targets on this pathway were further selected for animal experimental validation.

### 2.4. Ameliorative Effects of Beta-Myrcene in Insomniac Mice

**Figure 2:**
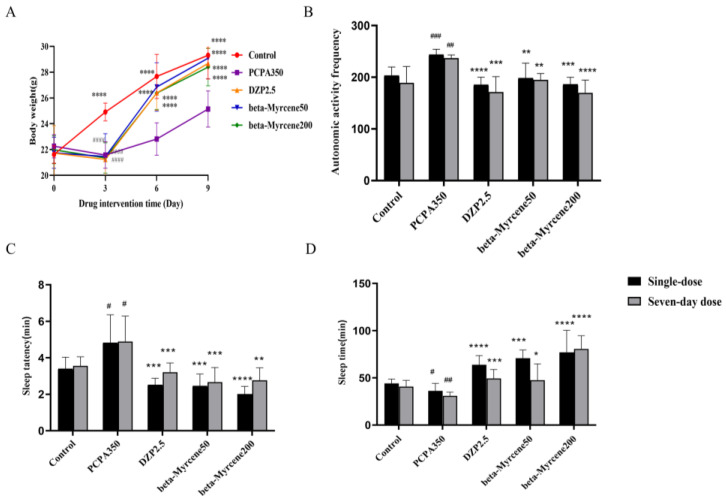
Effect of beta-myrcene on (**A**) body weights in PCPA-induced insomnia mice. (**B**) The mice’s autonomic activity frequency on the test after 30 min of dose administration. Suprathreshold anaobarbital sodium pentobarbital (85 mg/kg) induced (**C**) the sleep latency and (**D**) sleeping time were assessed. The treatment cycles of all mice were single-dose or seven-day doses. ^#^ *p* < 0.05, ^##^ *p* < 0.01, ^###^ *p* < 0.001 compared with control; * *p* < 0.05, ** *p* < 0.01, *** *p* < 0.001, **** *p* < 0.0001 compared with PCPA set. The results are presented as mean ± SD (*n* = 12).

The overall mental state of the mice was fine, with shiny fur, and they exhibited normal daily activities before being modeled. They were alert and not easily startled by external stimuli. The mice showed signs of poor mental state, increased activities, heightened irritability, and susceptibility to being startled after administration of PCPA. There was also an increase in aggressive behaviors such as fighting and attacking. Next, food intake was significantly reduced, with body weight notably lower than the blank control group (*p* < 0.0001). After treatment with both diazepam and beta-myrcene, groups showed a recovery in food intake and there was a significant increase in body weight (*p* < 0.0001), while the mice in the model group exhibited slower growth in body weight (Figure 2A).

### 2.5. Effects of Beta-Myrcene on Autonomous Activity in Insomniac Mice

In a new environment, the spontaneous activity, exploratory behavior, and stress levels of experimental animals undergo changes. Open-field test is commonly used to assess these alterations and is widely employed to evaluate behavioral changes in animals, including the presence of anxiety or depressive behaviors [17]. The mice in all groups except the blank control group were placed in an activity box after administration of diazepam or beta-myrcene, and the number of activities of mice was recorded (Figure 2B). Thirty minutes after the final treatment, the mice were placed in clean cages to acclimatize for 5 min before starting the test. The number of movements of the mice in the model group was significantly increased on the first day (*p* < 0.001) and the seventh day (*p* < 0.01) compared to the control group. A single administration of diazepam resulted in a significant decrease in the number of activities (single-dose: *p* < 0.0001; seven-day dose: *p* < 0.001), while the sedative–hypnotic effect of multiple administrations of diazepam was also decreased, which was consistent with previous reports [18,19]. The number of activities in the beta-myrcene group of mice was significantly reduced in a single dose of beta-myrcene (single-dose: *p* < 0.001; seven-day dose: *p* < 0.001) and decreased in a dose-dependent manner after seven days of administration. This indicated that beta-myrcene can increase the quiet state of mice. Interestingly, after multiple administrations of the low-concentration group of beta-myrcene, sedative effects were maintained without obvious desensitization compared to a single administration, and the high-concentration group had a better effect.

### 2.6. Effects of Beta-Myrcene on Anaobarbital Sodium-Induced Sleep Test in Mice

Anaobarbital sodium-induced sleep test is a commonly used pharmacological method to detect sedative and hypnotic effects of drugs. Anaobarbital sodium is a central nervous depressant, which has sedative and hypnotic effects and can shorten the sleep latency and prolong sleep duration in laboratory animals. The sleep latency and sleep duration of experimental animals were explored by using subthreshold dose and suprathreshold dose [20]. The rate of sleep on a subthreshold dose (50 mg/kg) of anaobarbital sodium is shown in Table 1. Single or seven-day administration of diazepam or beta-myrcene increased the subthreshold dose sleep rate of anaobarbital sodium compared with the model group, whereas the function of diazepam was reduced and beta-myrcene had a dose-dependent increase in its effect. Additionally, the hypnotic function of the mice was basically maintained after seven days of administration of beta-myrcene.

The sleep latency and sleep duration of a suprathreshold dose (85 mg/kg) of anaobarbital sodium are shown in Figure 2C,D. Compared to the model group, the administration of diazepam or beta-myrcene resulted in a shortened sleep latency and prolonged sleep duration. Notably, the sleep latency in mice was largely maintained after single or seven-day administrations of diazepam and in low-dosage groups of beta-myrcene (*p* < 0.001). It is worth noting that beta-myrcene increased sleep duration in a dose-dependent manner, both for single and seven-day administration, without exhibiting significant desensitization. However, the efficacy of diazepam decreased as the administration time increased. Beta-myrcene has the same effect as diazepam, since both have the ability to enhance the hypnotic effects of anaobarbital sodium by prolonging its sleep duration, decreasing sleep latency, and increasing the rate of falling asleep in insomnia mice, especially the beta-myrcene.

### 2.7. Effects of Beta-Myrcene on the Hypothalamus of Insomnia Mice

**Figure 3:**
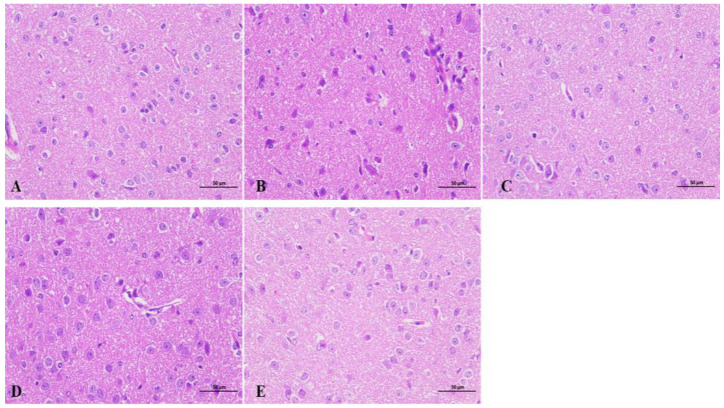
Effects of beta-myrcene on the hypothalamus of insomnia mice after seven days of administration. Hypothalamic sections were stained with hematoxylin and eosin (H&E stain 400×, *n* = 3). (**A**) Control group. (**B**) PCPA group. (**C**) Diazepam group. (**D**) Beta-Myrcene (50 mg/kg) group. (**E**) Beta-Myrcene (200 mg/kg) group.

As shown in Figure 3, the hypothalamus of mice on the seventh day of beta-myrcene administration was used for observation. Hypothalamic neuronal cells of the blank group had good morphology and were evenly distributed. The molecular layer and the outer granular layer were more clearly demarcated, with the nucleus clearly visible, large, and rounded, and the cells were full. In the model group, the hypothalamic neuronal cells were severely deformed, disordered, loose, and less compact, with numerous small broken cells, deeply stained nuclei, and ambiguous nucleoli. After administration of beta-myrcene or diazepam, the hypothalamic neuronal cells were significantly improved compared with the model group. As observed, neuronal cells are neatly arranged with clear boundaries, cell vacuoles, reduced nuclear consolidation phenomenon, intercellular distance restored to normal, nucleoli are obvious, morphology are full, and the effect was remarkable. It is suggested that beta-myrcene can reduce the damage of hypothalamic neuron cells induced by PCPA and has a protective effect on brain injury.

### 2.8. Effects of Beta-Myrcene on Neurotransmitter Levels in PCPA-Induced Insomnia Mice

**Figure 4:**
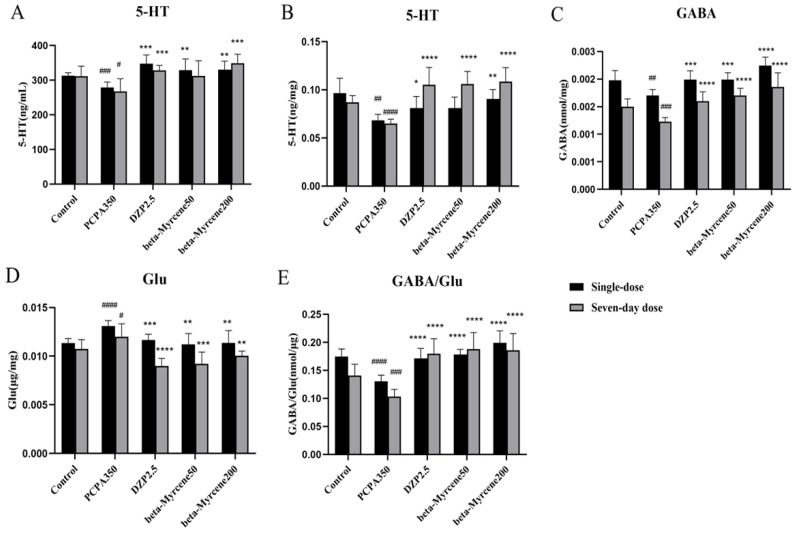
Effects of beta-myrcene on neurotransmitter levels in PCPA-induced insomnia mice after single dose or seven-day dose. Concentration of (**A**) 5-HT in mice serum. (**B**) 5-HT in mice hypothalamus. (**C**) GABA in mice hypothalamus. (**D**) Glu in mice hypothalamus. (**E**) GABA/Glu in mice hypothalamus. ^#^ *p* < 0.05, ^##^ *p* < 0.01, ^###^ *p* < 0.001, ^####^ *p* < 0.0001, compared with control; * *p* < 0.05, ** *p* < 0.01, *** *p* < 0.001, **** *p* < 0.0001, compared with PCPA set. The results are presented as mean ± SD (*n* = 12).

Neurotransmitters play a crucial role in nerve signaling, and many sedative and hypnotic drugs exert pharmacological effects by altering the concentration of neurotransmitters [21]. Therefore, ELISA kits were used to detect neurotransmitters in serum 5-HT and hypothalamus GABA, 5-HT, and Glu of insomnia mice (Figure 4A–E). The results showed that the neurotransmitter levels of 5-HT and GABA were reduced to varying degrees after the experimental model was applied, while Glu levels showed the opposite trend. After administration of diazepam or beta-myrcene, serum levels of 5-HT were significantly elevated in mice and basically maintained after single or seven-day administrations. Meanwhile, in the hypothalamus, the inhibitory neurotransmitter level of 5-HT was significantly increased after seven-day administrations compared with the model group (*p* < 0.0001). Beta-myrcene treatment significantly decreased the excitatory neurotransmitter level of Glu at 50 mg/kg (single dose: *p* < 0.01; seven-day dose: *p* < 0.001) and at 200 mg/kg (*p* < 0.01). The content of GABA/Glu was significantly increased (*p* < 0.0001) and basically maintained. It is suggested that beta-myrcene can indirectly improve insomnia by increasing neurotransmitters in the serum and hypothalamus of insomnia mice.

### 2.9. Effects of Beta-Myrcene on the Levels of SOD and MDA in PCPA-Induced Insomnia Mice Sera

**Figure 5:**
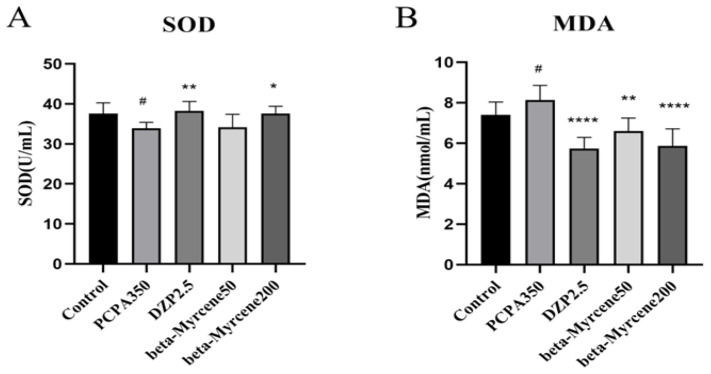
Effects of beta-myrcene on superoxide dismutase (SOD) and malondialdehyde (MDA) levels in serum of PCPA-induced insomnia rats after seven-day dose (*n* = 8). (**A**) SOD; (**B**) MDA. ^#^ *p* < 0.05 compared with control; * *p* < 0.05, ** *p* < 0.01, **** *p* < 0.0001, compared with PCPA set. The results are presented as mean ± SD (*n* = 12).

SOD is an important antioxidant enzyme in the body, which can be used to remove excess superoxide anion free radicals in the body. MDA is a substance produced by the peroxidation of lipids in the body. Therefore, the degree of damage to the body is often determined by measuring the content of SOD and MDA [22,23]. We also examined the effects of beta-myrcene on the levels of serum SOD and MDA after seven-day administration (Figure 5A,B). Respectively, SOD activity was significantly higher after administration of diazepam (*p* < 0.01) or a high dose of beta-myrcene (*p* < 0.05) than that in the insomnia mice after 7 days, while there was no significant change in the low dose of beta-myrcene. The opposite was true for MDA levels.

### 2.10. Effects of Beta-Myrcene on the 5-HT1AR, GABAARα1, GABAARγ2, and GluR1 mRNA Levels

**Figure 6:**
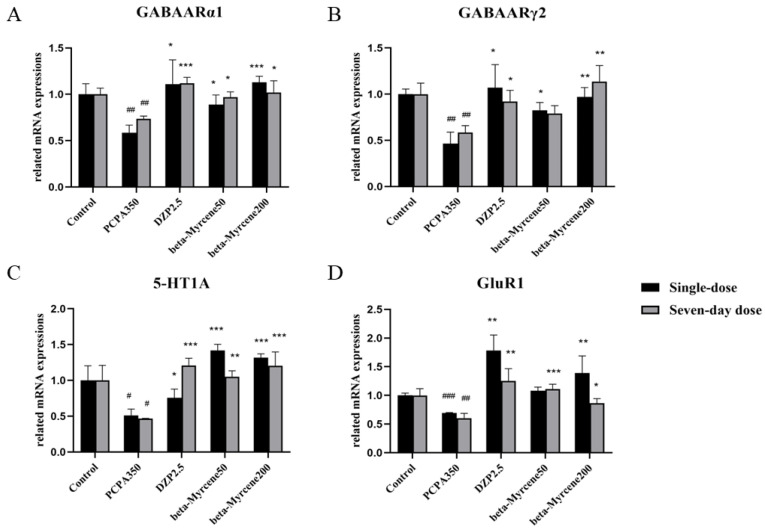
(**A**) Expression levels of GABAARα1 mRNA after treatment with beta-myrcene in the hypothalamus of mice. (**B**) Expression levels of GABAARγ2 mRNA after treatment with beta-myrcene in the hypothalamus of mice. (**C**) Expression levels of 5-HT1A mRNA after treatment with beta-myrcene in the hypothalamus of mice. (**D**) Expression levels of GluR1 mRNA after treatment with beta-myrcene in the hypothalamus of mice. The treatment cycles of all mice were single dose or seven-day doses. ^#^ *p* < 0.05, ^##^ *p* < 0.01, ^###^ *p* < 0.001, compared with control; * *p* < 0.05, ** *p* < 0.01, *** *p* < 0.001, compared with PCPA set. The results are presented as mean ± SD (*n* = 3).

Based on the above results, further research was conducted from the perspective of genes and RT-PCR was used to investigate the mRNA level of 5-HT1AR, GABAAR*α*1, GABAAR*γ*2, and GluR1 in the mice hypothalamus. As shown in Figure 6, GABAAR*α*1, GABAAR*γ*2, 5-HT1A, and GluR1 levels were significantly reduced in the model group mice compared to the control group. This reduction was attenuated by treatment with beta-myrcene at 50 and 200 mg/kg or diazepam at 2.5 mg/kg. The mRNA levels of several genes increased dose-dependently after administration of beta-myrcene.

### 2.11. Effects of Beta-Myrcene on the GAD65, GAD67, GABAARγ2, PKA, and 5-HT1A Proteins Levels

**Figure 7:**
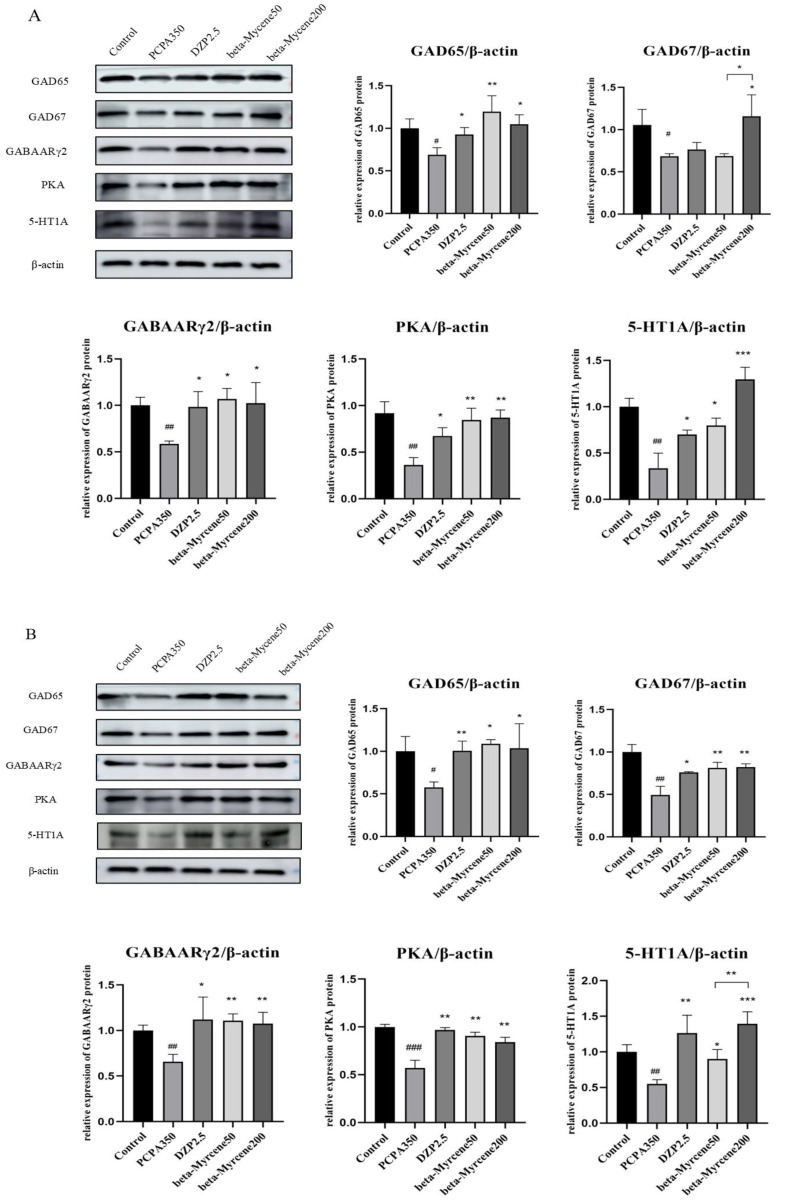
Effects of single or seven-day dose of beta-myrcene on protein expression in the hypothalamus. (**A**) The protein expression of GAD65, GAD67, GABAARγ2, PKA, and 5-HT1A was detected by Western blot in the hypothalamus of mice after a single dose. (**B**) The protein expression of GAD65, GAD67, GABAARγ2, PKA, and 5-HT1A was detected by Western blot in the hypothalamus of mice after a seven-day dose. ^#^ *p* < 0.05, ^##^ *p* < 0.01, ^###^ *p* < 0.001, compared with control; * *p* < 0.05, ** *p* < 0.01, *** *p* < 0.001, compared with PCPA set. The results are presented as mean ± SD (*n* = 3).

The expression levels of the proteins of GAD65, GAD67, GABAAR*γ*2, PKA, and 5-HT1A in the mice hypothalamus were determined by Western blot assay. In a single administration, the expression levels of GAD65, GAD67, GABAAR*γ*2, PKA, and 5-HT1A were significantly decreased in the model group compared with the control group. Compared to the model group, GAD65, GABAAR*γ*2, PKA, and 5-HT1A protein levels were differentially elevated after the administration of diazepam or beta-myrcene treatment. GAD67 protein levels were significantly increased in the high-dose beta-myrcene group and there was no significant change in the low-dose group (Figure 7A).

In the seven days of administration, GAD65, GAD67, GABAAR*γ*2, PKA, and 5-HT1A protein levels were differentially elevated in the diazepam group and in both doses of beta-myrcene group, and the results are shown in Figure 7B.

**Figure 8:**
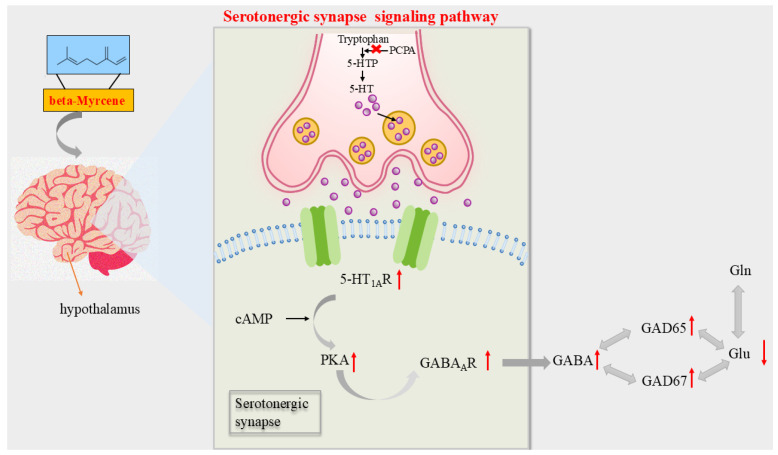
Diagram of the mechanism of action of beta-myrcene on insomnia mice. PCPA caused insomnia in mice by inhibiting 5-HTP. In the hypothalamus of PCPA-induced insomnia mice, beta-myrcene acted on the 5-HT1AR target by releasing the neurotransmitter, upregulating PKA and GABAAR, increasing levels of the GABA, and upregulating GAD65 and GAD67. It regulated the balance of GABA and GLU to improve insomnia. An upward arrow indicates an upward arrow, and a downward arrow indicates a downward arrow.

According to the above results, beta-myrcene, the active ingredient of LEO, can improve the symptoms of PCPA-induced insomnia in mice. Furthermore, mechanistic studies supported that beta-myrcene acted as a treatment for insomnia through the serotonergic synaptic pathway and indirectly mediated sleep by maintaining the balance of Glu and GABA, and the graphic abstract is shown in Figure 8.

## 3. Discussion

Lavender is a well-known aromatic plant in the world for treating insomnia. It is effective in regulating insomnia by modulating the serotonergic signaling pathway [24]. However, there is insufficient evidence to support the anti-insomnia activity of LEO in identifying its active components and potential targets. Therefore, the main purpose of this study is to explore the possible “Active Ingredients-Potential Targets-Pathways” of LEO through network pharmacology. Based on the literature, 15 LEO terpenes were screened as research subjects, and 128 potential targets of LEO for sleep regulation were found, accounting for 76.65% of the total active ingredient targets, suggesting that LEO might exert its potential pharmacological effects on insomnia through multiple targets. The results of GO and KEGG enrichment analyses further showed that LEO might display anti-insomnia activity by mediating the typical serotonergic synaptic signaling pathway and cAMP signaling pathway. Beta-myrcene was selected as the main active component. The sedative and hypnotic effects of beta-myrcene through the serotonergic synaptic signaling pathway were verified through animal experiments. In this paper, PCPA was used to induce insomnia in mice. In the context of insomnia, PCPA, as a tryptophan hydroxylase inhibitor, reduced 5-HT levels in the brain, thereby exacerbating insomnia [25]. Compared to normal animals, mice with intraperitoneal-injected PCPA exhibit a significantly higher level of aggression, as well as lower food intake and weight. This result was consistent with the literature findings suggesting that PCPA can lead to dysbiosis in the gut microbiota and metabolic disruption [26]. Mice administered with diazepam or beta-myrcene were able to maintain a healthy diet and gain weight, leading to improved mental state. These results indicated that beta-myrcene can ameliorate the adverse effects caused by PCPA. The anaobarbital sodium sleep test was used as a common pharmacodynamic study method to test whether the drug has a hypnotic effect, whose mechanism of action is mainly related to the blockade of the upward activation system of the brainstem network structure. Administering a threshold dose of anaobarbital sodium induced the disappearance of the righting reflex in mice, allowing for the observation of the synergistic effects on sleep [27]. In order to avoid the effects of heparanase inhibitors, subthreshold experiments should be performed first. Beta-myrcene increased the sleep rate in mice after administration as a dose-dependent process and maintained the effect of a single dose or seven days of dosing. However, the efficacy of diazepam decreased with the increase in administration time. Upon administration of a suprathreshold dose of anaobarbital sodium, beta-myrcene not only significantly shortened sleep latency, rapidly induced sleep, and significantly prolonged sleep duration in PCPA insomniac mice but also had a stable effect with single or multiple administrations. These results suggested that beta-myrcene can synergize with anaobarbital sodium to achieve a sedative–hypnotic effect.

The hypothalamus plays a crucial role in regulating the sleep–wake cycle. During sleep, the hypothalamus deactivates the arousal system and stabilizes this behavioral state [28]. Pathological observation of the hypothalamus in mice showed that, compared with the control group, the hypothalamic neuronal cells in the PCPA group were disorganized and loosely arranged and not tightly packed, with many broken small cells and blurred nucleoli. The administration of beta-myrcene or diazepam significantly reversed the damage caused by PCPA, and the neuronal cells were full of morphology, neatly aligned, and with clear boundaries, which was a remarkable effect. Therefore, the hypothalamic H&E staining results indicated that beta-myrcene can repair the damage to neuronal cells caused by PCPA.

Neurotransmitters play a critically important role in information processing throughout the nervous system [29]. In the central nervous system, 5-HT and GABA are the primary inhibitory neurotransmitters, while Glu is the main excitatory neurotransmitter. Serotonin, also known as 5-HT, is an important monoamine neurotransmitter in the central nervous system. The cell bodies of serotonergic neurons in the brain are primarily located in the nucleus accumbens and played a role in regulating and maintaining the sleep–wake cycle, mainly promoting arousal and inhibiting REM sleep [30]. In turn, 5-HT inhibits orexin neurons by activating 5-HTR1A, which has the ability to promote arousal and maintain and stabilize behavioral states [31]. It is shown that insomniac mice develop a significant reduction in 5-HT levels and 5-HTR1A protein expression [32]. GABA is a major inhibitory neurotransmitter in the nervous system and can be involved in sleep regulation; GABA levels can be used as a marker of hypnotic effects [33]. The glutamatergic system includes the excitatory neurotransmitter Glu. Therefore, we mainly measured the neurotransmitter levels of GABA, 5-HT, and Glu. Compared with the model group, the 5-HT, GABA, and GABA/Glu contents were enhanced in beta-myrcene-treated insomnia mice administered on a single or seven days, and the Glu content was significantly decreased. Based on these results, beta-myrcene can be used to treat insomnia by inhibiting the excitability of the central nervous system and regulating the concentrations of various neurotransmitters.

Additionally, oxidative stress is associated with insomnia, which can also regulate the sleep–wake circadian rhythm such as SOD and MDA [34]. In normal cells, the major antioxidant enzyme SOD is an important scavenger of harmful ROS, excess of which leads to oxidative stress and lipid peroxidation [23]. MDA is a substance obtained by peroxidation of lipids in the organism, which indirectly reflects the severity of free radical attack on the cells of the organism. In this study, when PCPA insomniac mice were intraperitoneally injected with diazepam or beta-myrcene, SOD activity was significantly increased, while that of MDA content was significantly reduced. Thus, beta-myrcene can reduce PCPA-induced oxidative stress in mice by increasing the activity of the antioxidant enzyme SOD and reducing the body’s malondialdehyde MDA content.

To further investigate the effects of beta-myrcene on molecular mechanisms, we used *RT-PCR* to detect the expression levels of mRNA at the gene level in the mice’s hypothalamus. Administration of diazepam significantly increased the expression of GABAAR*α*1, GABAAR*γ*2, 5-HT1A, and GluR1. One day and seven days after administration of beta-myrcene also significantly increased the expression of the above four genes, and the results suggested that beta-myrcene may be involved in the regulation of sedative–hypnotic hypnosis by increasing the gene expression of GABAAR*α*1, GABAAR*γ*2, 5-HT1A, and GluR1. Simultaneously, beta-myrcene may have a different regulatory effect compared to diazepam. Reports indicated that tolerance to the sedative effects of benzodiazepines is associated with abnormal expression of GABAA receptors [18,35]. Correlation analysis was conducted to elucidate the interconnection between alterations in the protein expression levels and sleep. We have measured the protein expression levels of 5-HT1A, PKA, and GABAAR within the serotonergic synaptic signaling pathway. Western blot results showed that administration of diazepam or beta-myrcene for one day and seven days significantly increased the expression of GAD65, GAD67, PKA, GABAAR*γ*2, and 5-HT1A proteins compared to the model group. In the serotonergic synaptic signaling pathway, 5-HT acts as a signaling molecule that binds to G-protein-coupled receptors on the surface of the cell membrane, triggering adenylate cyclase, which produces the second messenger cyclic adenosine monophosphate (cAMP), which promotes sleep through activation of protein kinase A (PKA) and acted to increase the levels of GABA [7]. Under the control of the rate-limiting enzymes glutamate dehydrogenase GAD65 and GAD57, Glu decarboxylated to GABA. When presynaptic neuronal vesicles release GABA into the synaptic gap, a metabolic cycle of GLU/GABA to Gln is formed, and the homeostasis of GABA and Glu together maintains sleep stability [29]. It is suggested that beta-myrcene can promote sleep through the serotonergic synaptic signaling pathway and indirectly play a sedative–hypnotic role by mediating the balance of GABA and Glu. Collectively, the in vivo results confirmed the network pharmacology results.

In summary, our study results demonstrated that beta-myrcene, one of the terpenes of LEO, exhibited significant anti-insomnia activity through network pharmacology. The in vivo effects included prolonging its sleep duration, decreasing sleep latency, and enhancing the rate of falling asleep and neurotransmitters. Furthermore, the mechanistic studies also suggested that beta-myrcene can exert therapeutic effects through the serotonergic synaptic signaling pathway. Meanwhile, beta-myrcene could participate in the balance of GABA and Glu to indirectly mediate sleep and play a sedative and hypnotic role. These findings provided the scientific basis for further experimental study on the effect of LEO on insomnia.

## 4. Materials and Methods

### 4.1. Animals

A total of 120 healthy male ICR mice weighing 18–22 g each, of SPF grade, were acquired from Henan SCXK Biotechnology Co., LTD (License No.: SCXK 2020-0005, Zhengzhou, Henan, China). The animals were fed adaptively in a sterile SPF laboratory for 3 days prior to the experiment. The room maintained a temperature of 25 °C, humidity of 55 ± 5%, and a photoperiod of 12 h light and 12 h darkness. The animals had free access to food and drink during this time. The animal research was approved by the Institutional Animal Care and Use Committee (IACUC) and adheres to institutional rules for animal welfare and experimental behavior.

### 4.2. Drugs and Drug Administration

Anaobarbital sodium was purchased from Shanghai Shangyao Xinya Pharm Co., H31021725, Shanghai, China. Diazepam was purchased from Shandong Sine Pharm Co., H37023039, Heze, China. Beta-myrcene was purchased from Chengdu Standard Sample Biotechnology Co., wkq22032504., Chengdu, China, and PCPA was purchased from Shanghai Aladdin Biochemical Technology Co., C2208165, Shanghai, China.

PCPA was suspended in mildly alkaline saline with 2% Tween 80 for abdomen injection [36]. Beta-myrcene or diazepam were, respectively, suspended in saline with 2% Tween80 and 2% DMSO. Anaobarbital sodium was dissolved in saline [21].

### 4.3. Network Pharmacology Analysis Based on “Component-Target”

#### 4.3.1. Acquisition of Active Ingredients of LEO Targets and Insomnia Disease Targets

**Figure 9:**
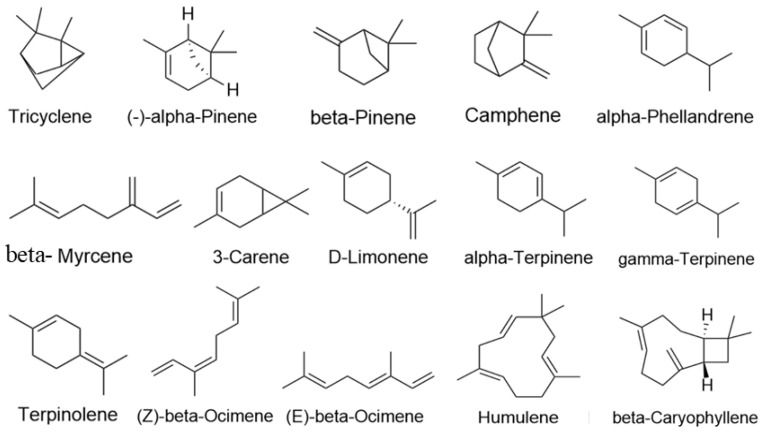
Active constituents structure of LEO.

In total, 15 major active ingredients in LEO were selected [16,37], including 13 monoterpenes and 2 sesquiterpenes, and ingredient types are detailed in Table 2; the 2D structure of its terpenes is shown in Figure 9. Swiss Target Prediction database [38] (http://swisstargetprediction.ch/, accessed on 9 January 2022) was used, “Homosapiens” was selected as a species, and Probability > 0 was used as a screening condition to process the results.

Then, the PharmMapper database (http://lilab-ecust.cn/pharmmapper/, accessed on 9 January 2022) [39] was used for potential target identification. The Retrieve/ID mapping feature of the Uniprot database (https://www.uniprot.org/uploadlists/, accessed on 9 January 2022) was utilized to translate the Uniplot in the target results into gene names. The retrieval results from both databases were merged, eliminating duplicates, to obtain the target database for the active components in LEO.

Gene targets associated with sleep disorders were identified by searching the GeneCards database [40] (https://www.uniprot.org/uniprot/, accessed on 9 January 2022), OMIM database [41] (https://omim.org/, accessed on 9 January 2022), and TTD database [42] (http://db.idrblab.net/ttd/, accessed on 9 January 2022) using the terms “Insomnia” and “Disorders of Initiating and Maintaining Sleep.” The health condition target library required for this investigation was obtained by consolidating the results from the three databases and subtracting any duplicate values.

The active ingredient library of LEO was combined with the sleep diseases target library to locate prospective illness targets through the elimination of the intersection.

#### 4.3.2. GO Analysis and KEGG Pathway Enrichment Analysis

The DAVID Bioinformatics Resources 6.8 database [43] (https://david.ncifcrf.gov/, accessed on 15 February 2022) was applied for a bioinformatics study to pinpoint the probable targets of the substances, with “OFFICIAL GENE SYMBOL” chosen as the identifier item. Once the species “Homo sapiens” and type “Gene List” were selected, the list was submitted for GO enrichment analysis and KEGG pathway enrichment analysis with an EASE value of 0.05 (*p* < 0.05); data related to CC, MF, BP, and KEGG pathways were obtained. The data were evaluated based on the enrichment scores and visual representations were created using the GO enrichment histogram and KEGG bubble map on the bioinformatics web platform (http://www.bioinformatics.com.cn/, accessed on 15 February 2022).

#### 4.3.3. Construction of the “Active Ingredient-Potential Target-Pathway” Network of LEO

The 15 selected LEO active ingredients and their target proteins were combined with signaling pathways from KEGG enrichment analysis and related targets associated with the pathways. These data were imported into Cytoscape 3.7.2 software to construct a “component-target-pathway” network visualization. NetworkAnalyzer was applied to assess the primary active ingredients and key targets in the network through the parameters of Degree, Betweenness, and Closeness to discover possible action pathways.

### 4.4. Experimental Verification

#### 4.4.1. Animal Model Preparation and Grouping

Once 120 ICR mice had undergone three days of adaptive feeding, they were assigned at random to the following groups: a blank control group, a PCPA group (350 mg/kg), a diazepam group (2.5 mg/kg), a beta-myrcene low-dose group (50 mg/kg), and a beta-myrcene high-dose group (200 mg/kg). A grand total of five groups were established. Within each group, 12 animals were subjected to single-dose observation and 12 animals were observed over a seven-day period of dosage. The blank group received an injection of physiological saline with Tween 80, while the other groups were injected with PCPA at a dosage of 350 mg/kg for three consecutive days to establish a model [44]. The PCPA group received an injection of physiological saline with Tween 80 after the model. Groups were given doses injected intraperitoneally once a day for seven days. The injection volume is 0.1 mL/10 g.

**Figure 10:**
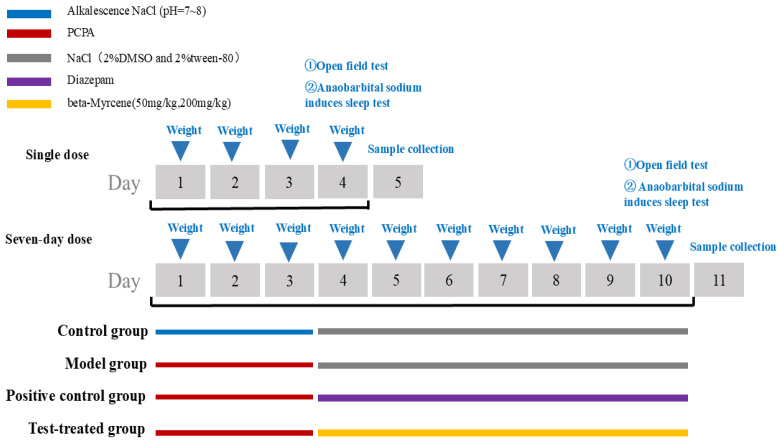
The procedure of the study on PCPA-induced insomnia mice.

The detailed procedure of the animal study is shown in Figure 10.

#### 4.4.2. Behavioral Observations in Mice

After the mice were grouped according to Section 4.4.1, 10 mice were taken from each group and placed in the animal autonomous activity apparatus (Anhui Zhenghua Biological Instrument Equipment Co., LTD, ZH-YLS-1C, Huaibei, China) for behavioral observation, respectively. After 5 min of acclimatization, the recorder was turned on to analyze and record the number of mice activities in each group within 10 min after the last 30 min of the first day and the seventh day of administration. The interval between the observation experiments for each group was observed by using disinfectant alcohol to wipe down the field box and clean the bottom of the box of the fecal matter. The surrounding environment was kept quiet during the test [33].

#### 4.4.3. Anaobarbital Sodium-Induced Sleep Test in Mice

At 30 min after the last dose, each group of mice was injected intraperitoneally with a subthreshold dose of 50 mg/kg of anaobarbital sodium. After the injection, the mice were placed on a warming pad and the sleep time was recorded. The number of mice in each group that fell asleep within 30 min was recorded using the disappearance of the turning reflex at 30 s after drug administration as an indicator of sleep onset, and the sleep rate of mice in each group was calculated. Sleep rate = number of mice falling asleep/total number of mice in each group [45].

The steps for the suprathreshold dose (85 mg/kg) were the same as above. Sleep latency and sleep duration of mice were recorded.

#### 4.4.4. Sample Collection

At the end of the experiment, all mice described under Section 4.4.1 were euthanized with 5% chloral hydrate and blood was taken from the abdominal aorta, centrifuged at 3000 rpm at 4 °C for 10 min, and rested for 2 h; the supernatant was aspirated and stored at −20 °C. After euthanasia of mice, the whole brains of three mice in each group were extracted and fixed in 4% paraformaldehyde and the hypothalamus was extracted from the rest of the mice, quick-frozen in liquid nitrogen, and then quickly transferred to −80 °C for storage.

#### 4.4.5. Histopathological Examinations (HE)

The hypothalamic lesions were histologically examined and identified by observing hematoxylin–eosin (H&E) staining. Whole brains of three mice from each group administered for seven days as described in Section 4.4.4 were fixed with 10% paraformaldehyde at 4 °C for 12 h. The tissue samples were dehydrated with different concentrations of ethanol, embedded in paraffin, and then sliced to a thickness of 5 μm. The sections were stained with hematoxylin and eosin and the pathological changes were observed under a 400× light microscope [26].

#### 4.4.6. Neurotransmitter Content Was Detected by ELISA

The levels of neurotransmitter 5-HT in mice serum and hypothalamus were detected by mouse 5-HT ELISA Kit (Wuhan bioswamp Biotechnology Co., LTD, MU30036, Wuhan, China), and the levels of neurotransmitters GABA and Glu in mice hypothalamus were detected by mouse GABA ELISA Kit (Wuhan bioswamp Biotechnology Co., LTD, MU30278, Wuhan, China) and mouse GLU ELISA Kit (Shanghai EK-Bioscience Biotechnology Co., LTD, EK- M27909, Shanghai, China). The hypothalamus sample described in Section 4.4.4 was weighed, added to PBS in the ratio mouse of 1:19, homogenized, centrifuged to obtain the supernatant, and a double antibody sandwich assay was carried out according to the instructions of the kit [46].

#### 4.4.7. Antioxidant Enzyme Activity Measurements

The levels of SOD and MDA in the serum of mice administered for seven days were measured by using a superoxide dismutase (SOD) kit (Nanjing Jiancheng Bioengineering Institute Co., LTD, A001-3-2, Nanjing, China) and malondialdehyde (MDA) kit (Nanjing Jiancheng Bioengineering Institute Co., LTD, A003-1-1, Nanjing, China) to investigate the antioxidant effects of beta-myrcene on PCPA-induced insomnia in mice.

#### 4.4.8. Real-Time Polymerase Chain Reaction (Rt-PCR)

Total RNA was extracted from the hypothalamus using Trizol reagent. The total RNA concentration was determined and then reverse-transcribed into cDNA. The cDNA was used as a template for quantitative real-time PCR analysis, which was performed using SYBR Green Realtime PCR Master Mix (TOYOBO Life Science Co., LTD, 238000, Shanghai, China). The data were analyzed using the BIO-Rad CFX Manager3.1 Software [7]. The primers used are shown in Table 3.

#### 4.4.9. Western Blot Analysis

The hypothalamus tissue samples were lysed and homogenized in cold RIPA (Beijing Labgic Technology Co., LTD, BL504A, Beijing, China) and cocktail (Beijing Labgic Technology Co., LTD, BL612A, Beijing, China). The solution was then centrifuged at 12,000 rpm for 10 min at 4 ℃ and the protein concentration in the supernatant was determined using a commercial BCA assay kit (Thermo Fisher Scientific Co., LTD, #23225, St. Bend, OR, America). Protein samples (50 μg) were separated by adding them to a sodium dodecyl sulfate (10%) gel and transferring to a 0.22 µm polyvinylidene difluoride (PVDF) membrane. After incubation with 5% skimmed milk powder for 2 h at room temperature, the primary antibody was incubated overnight at 4 °C. Subsequently, TBST was used to wash the membrane and then it was incubated at room temperature for 1 h with horseradish peroxidase coupled with a secondary antibody. Rabbit anti-5HT1A (Affinit, AF5453, 1:1000), rabbit anti-GABA*γ*2 (Affinit, DF6583, 1:1000), rabbit anti-GAD65 (ABclonal, A22728, 1:5000), rabbit anti-GAD67 (ABclonal, A1475, 1:1000), and rabbit anti PKA (Affinit, AF5450, 1:1000) were used as primary antibodies. Mouse anti-*β*-actin (Affinit, AF7018, 1:1000) was used as an internal reference for *β*-actin expression. Finally, the membranes were washed with TBST and the band intensities were visualized using the ECL reagent (Abbkine, ATWI19081). A gel image was used (AlphaImager System) to analyze the target bands and calculate the relative expression of Western blotting data.

#### 4.4.10. Statistical Analyses

The data were analyzed using GraphPadPrism8 statistical software. Differences between the groups were examined using Student’s *t*-test or one-way ANOVA analysis and *p* < 0.05 was considered to be a statistically significant difference among the groups.

**Figure 11:**
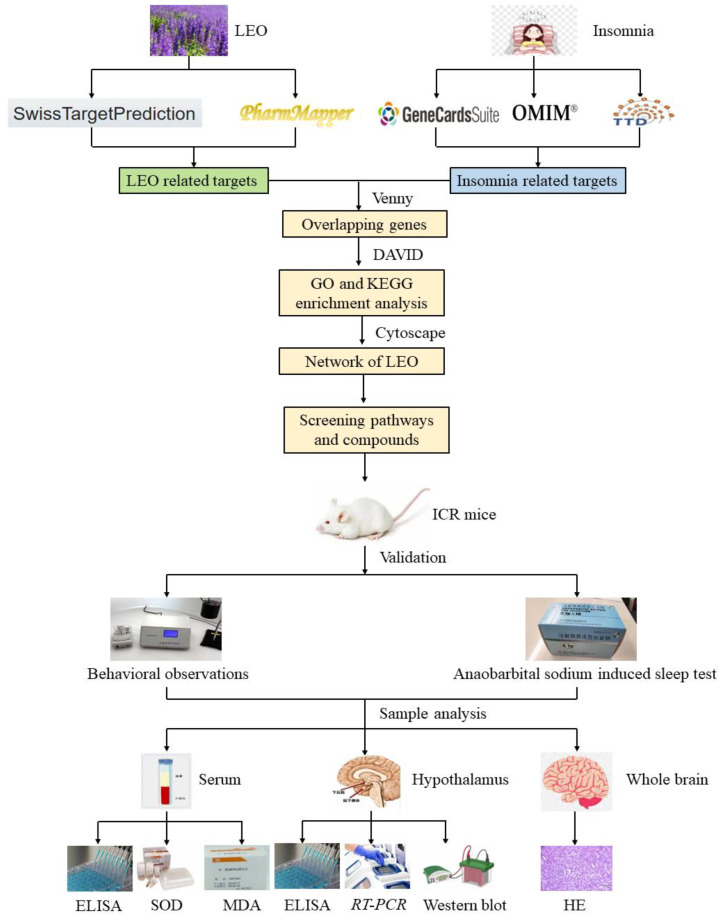
The flow diagram illustrates network pharmacological analysis methods and validation strategies for in vivo and in vitro experiments. ELISA: enzyme-linked immunosorbent assay; SOD: superoxide dismutase; MDA: malondialdehyde; RT-PCR: real-time polymerase chain reaction; HE: histopathological examinations.

The workflow of the integrated systems pharmacology approach employed herein is illustrated in Figure 11.

## 5. Conclusions

In this study, beta-myrcene was screened as the main active ingredient in LEO through network pharmacological analysis. The most classic serotonergic synapse signaling pathway was then selected for further investigation. Experimental research has shown that beta-myrcene can play a sedative and hypnotic role by mediating insomnia-related genes and target proteins, affecting serotonergic synaptic signaling pathway, increasing the quiet state of mice, reducing sleep latency, increasing sleep duration, and increasing the content of neurotransmitters in mice, as well as alleviating oxidative stress and neuronal cell damage caused by PCPA. Through the method of network pharmacology combined with experimental verification, this study elucidated the potential pharmacological mechanism underlying the use of LEO in the treatment of insomnia. The findings provide a reference for the future clinical application of LEO.

## Figures and Tables

**Table 1:** Effects of beta-myrcene on the subthreshold anaobarbital sodium-induced hypnosis.

			Single Dose		Seven-Day Dose	
Group	Dose (mg/kg)	Total Number	Sleeping Mice	Sleeping Rate (%)	Sleeping Mice	Sleeping Rate (%)
Control	-	12	3	25	3	25
PCPA	350	12	1	8.33	1	8.33
Diazepam	2.5	12	8	66.67	5	41.67
beta-Myrcene	50	12	6	50	6	50
beta-Myrcene	200	12	12	100	11	91.67

Sleep was induced in mice by subthreshold doses of anaobarbital sodium (50 mg/kg) after administration of saline (control), PCPA (350 mg/kg), beta-myrcene (50, 200 mg/kg), and diazepam (2.5 mg/kg) at a single or seven-day dose. Diazepam as a positive drug in co-ordination with anaobarbital sodium could induce sleep (*n* = 12).

**Table 2:** Active constituents of LEO.

Active Ingredients	CAS	Component Type
Tricyclene	508-32-7	Monoterpene
(-)-alpha-Pinene	7785-26-4	Monoterpene
beta-Pinene	127-91-3	Monoterpene
Camphene	79-92-5	Monoterpene
alpha-Phellandrene	99-83-2	Monoterpene
beta-Myrcene	123-35-3	Monoterpene
3-Carene	13466-78-9	Monoterpene
D-Limonene	5989-27-5	Monoterpene
alpha-Terpinene	99-86-5	Monoterpene
gamma-Terpinene	99-85-4	Monoterpene
Terpinolene	586-62-9	Monoterpene
(Z)-beta-Ocimene	3338-55-4	Monoterpene
(E)-beta-Ocimene	3779-61-1	Monoterpene
Humulene	6753-98-6	Sesquiterpene
beta-Caryophyllene	87-44-5	Sesquiterpene

**Table 3:** The primer sequence in *Rt-PCR*.

Mouse	**Gene**	**Primers**	**Size (bps)**
	GABAARγ2	F 5′ AGAATATGGCTATGAGTGTTTGGATGG 3′	27
		R 5′ GGCTCCTGTTCGGCAATCTTC 3′	21
	GABAARα1	F 5′ CCGTTCAGTGGTTGTAGCAGAAG 3′	23
		R 5′ TTCAAGTGGAAGTGAGTCGTCATAAC 3′	26
	5-HT1A	F 5′ TTCTATATTCCGCTGCTGCTCATG 3′	24
		R 5′ CCACCTTCTTGACCGTCTTGC 3′	21
	GLUR1	F 5′ ACAACTCAAGCGTCCAGAATAGAAC 3′	25
		R 5′ CCTCATAGCGGTCATTGCCTTC 3′	22
	β-actin	F 5′ GAGGGAAATCGTGCGTGAC 3′	19
		R 5′ GCTGGAAGGTGGACAGTGAG 3′	20

## Data Availability

Data will be made available upon request.

## Abbreviations

OMIM	Online Mendelian Inheritance in Man
TTD	Therapeutic Target Database
PCPA	DL-4-chlorophenylalanine
LEO	Lavender essential oil
GO	Gene Ontology
KEGG	Kyoto Encyclopedia of Genes and Genomes
5-HT	5-hydroxytryptamine
GABA	gamma-aminobutyric acid
PKA	protein kinase A
GLU	Glutamic acid
CC	cellular components
MF	molecular functions
BP	biological processes
HE	Histopathological examinations
ELISA	Enzyme-linked immunosorbent assay
SOD	Superoxide dismutase
MDA	Malondialdehyde
Rt-PCR	Real-time polymerase chain reaction
ECL	Electroche miluminescence
GAD67	glutamate decarboxylase 67
GAD65	glutamate decarboxylase 65
5-HT1AR	5-hydroxytryptamine receptor 1A
PVDF	polyvinylidene difluoride
